# Collagen Type III as a Possible Blood Biomarker of Fibrosis in Equine Endometrium

**DOI:** 10.3390/ani12141854

**Published:** 2022-07-21

**Authors:** Joana Alpoim-Moreira, Carina Fernandes, Maria Rosa Rebordão, Ana Luísa Costa, Miguel Bliebernicht, Telmo Nunes, Anna Szóstek-Mioduchowska, Dariusz J. Skarzynski, Graça Ferreira-Dias

**Affiliations:** 1 CIISA—Center for Interdisciplinary Research in Animal Health, Faculty of Veterinary Medicine, University of Lisbon, 1300-477 Lisbon, Portugal; joanaalpoimmoreira@hotmail.com (J.A.-M.); fachica@hotmail.com (C.F.); milorebordao@gmail.com (M.R.R.); tnunes@fmv.ulisboa.pt (T.N.); 2 Associate Laboratory for Animal and Veterinary Sciences (AL4AnimalS), 1300-477 Lisbon, Portugal; 3 Polytechnic of Coimbra, Coimbra Agriculture School, Bencanta, 3045-601 Coimbra, Portugal; 4 Embriovet—Prestação de Serviços Veterinários Lda., 2125-348 Muge, Portugal; analu.costa86@gmail.com (A.L.C.); miguel@embriovet.pt (M.B.); 5 Institute of Animal Reproduction and Food Research, Polish Academy of Sciences, 10-748 Olsztyn, Poland; a.szostek-mioduchowska@pan.olsztyn.pl (A.S.-M.); skadar@pan.olsztyn.pl (D.J.S.)

**Keywords:** collagen, biomarker, blood, endometrium, fibrosis, mare, endometriosis

## Abstract

**Simple Summary:**

In the mare, endometrosis is a disease characterized by excessive collagen fibers deposition in the endometrium (uterus inner layer), which is responsible for infertility. The gold standard method for endometrosis evaluation has been endometrial biopsy histopathological classification. The use of blood biomarkers for endometrosis identification would be less invasive, and could provide additional information regarding endometrosis diagnosis and fertility prognosis. Therefore, this study aimed to identify possible blood biomarkers for endometrosis diagnosis and fertility assessment on mares. Reproductive examination, endometrial biopsy histopathological classification, and blood collection were performed. Endometrium and serum collagen type I (COL1) and type III (COL3), and hydroxyproline concentrations were determined. In conclusion, serum COL3 concentration might be considered as a potential aid for the diagnosis of endometrosis and fertility prognosis in the mare. In contrast, COL1 and hydroxyproline did not prove to be effective as biomarkers of endometrial fibrosis in this species. Although it is very unlikely that a single blood biomarker could replace a histopathological evaluation, serum COL3 may have clinical applications. Thus, it may be useful to evaluate a group of mares as possible recipients in embryo transfer programs, where performing endometrial biopsies of several mares is not feasible.

**Abstract:**

Collagen pathological deposition in equine endometrium (endometrosis) is responsible for infertility. Kenney and Doig’s endometrial biopsy histopathological classification is the gold standard method for endometrosis evaluation, whereby blood biomarkers identification would be less invasive and could provide additional information regarding endometrosis diagnosis and fertility prognosis. This study aimed to identify blood biomarkers for endometrosis diagnosis (42 mares were used in experiment 1), and fertility assessment (50 mares were used in experiment 2). Reproductive examination, endometrial biopsy histopathological classification (Kenney and Doig) and blood collection were performed. Endometrium and serum collagen type I (COL1) and type III (COL3), and hydroxyproline concentrations were measured (ELISA). Serum COL3 cut-off value of 60.9 ng/mL allowed healthy endometria (category I) differentiation from endometria with degenerative/fibrotic lesions (categories IIA, IIB or III) with 100% specificity and 75.9% sensitivity. This cut-off value enabled category I + IIA differentiation from IIB + III (76% specificity, 81% sensitivity), and category III differentiation from others (65% specificity, 92.3% sensitivity). COL1 and hydroxyproline were not valid as blood biomarkers. Serum COL3 cut-off value of 146 ng/mL differentiated fertile from infertile mares (82.4% specificity, 55.6% sensitivity), and was not correlated with mares’ age. Only COL3 may prove useful as a diagnostic aid in mares with endometrial fibrosis and as a fertility indicator.

## 1. Introduction

Endometrosis, a term first introduced by Kenney in 1992 [1], is one of the causes of infertility in mares. Endometrosis is mainly characterized by periglandular fibrosis of the endometrium [2,3], which compromises the integrity and function of the endometrial glands required for embryo survival in the preimplantation period and for placental development [4]. The degree of endometrosis in mares increases with age [3,5,6], even though it is thought not to be associated with the number of foalings [7,8,9]. Atypical morphological and functional differentiation of periglandular endometrial stromal cells is the first sign of endometrosis. The first stage of fibrosis is characterized by large, polygonal periglandular stromal cells, which synthesize collagen fibers, whereas in advanced fibrosis, without signs of collagen synthesis [10], there is a predominance of metabolic active or inactive stromal cells, as well as myofibroblasts [8,10,11,12,13]. Myofibroblasts, which are fibroblast-derived cells, have been recognized as the main source of type I collagen (COL1) and of fibrogenic/inflammatory cytokines in fibrotic lesions [14,15]. The type of collagen present in endometrosis characterizes the chronology of this condition. In repair and fibrotic processes, collagen type III (COL 3) is the first to be expressed, followed by its replacement by COL1 in the extracellular matrix (ECM) [16,17]. Most degenerative changes typical for endometrosis can be diagnosed only through the histological evaluation of an endometrial biopsy [7,9,18,19,20,21,22,23,24,25,26]. Endometrial biopsy has been the gold standard for evaluation of the health of the mare’s uterus for over 50 years. Currently, the fertility prognosis is based on the categorization scale of Kenney and Doig [19], together with amendments of Schoon [3,5]. Even though it has been considered as a safe, practical, and especially useful method [18,21], and as an essential part of the breeding standard examination, it does not provide a 100% accurate information, as the biopsy sample taken may not represent the state of the whole uterus. The search for an additional and less invasive technique would be desirable. Therefore, the use of a blood biomarker should be considered. One key consideration in the assessment of any biomarker is the biological likelihood of its relationship to the pathological or physiological condition it measures. The use of collagen fragments for the assessment of fibrotic disorders is now well established in humans [27]. Blood biomarkers have been studied in horses to assess musculoskeletal conditions such as osteochondrosis [28,29,30,31], and some types of collagens have been used in humans for diagnostic purposes [27,32,33]; thus, the rationale of the present study was to find a putative correlation between endometrial fibrosis and blood COL, to be used as a diagnostic blood marker of endometrosis in mares. Due to the highly restricted distribution of hydroxyproline in collagen and elastin, the hydroxyproline content generally reflects the amount of collagen in samples. Therefore, since quantification of hydroxyproline has been utilized for evaluating tissue fibrosis or collagen deposition [34,35,36], it was used in this study. As the degree of endometrosis in mares increases with age [3,5,6], the age effect was also studied. Thus, we hypothesized that COL1, COL3 or hydroxyproline could be used as blood biomarkers of endometrial fibrosis in the mare. As such, we aimed to investigate the concentrations of COL1, COL3 and hydroxyproline in blood serum and endometria from mares with different endometrial categories, graded according to the histopathological system of Kenney and Doig [19] in experiment 1, and with fertility outcome in experiment 2.

## 2. Materials and Methods

### 2.1. Experimental Design

The present work included two different experiments. In experiment 1, venous blood and endometrial biopsies were obtained from the same mares for determination of COL1, COL3 and hydroxyproline concentrations, both in serum and in endometrial tissue graded according to Kenney and Doig’s classification [19]. Experiment 2 was conducted in different mares, after conclusion of experiment 1. Only venous blood samples were retrieved from those mares, later determined as fertile or infertile. In experiment 2, only COL3 was assessed based on experiment 1 results. All the procedures complied to welfare mandates, authorized by the Ethic Committee for Research and Teaching (Comissão de Ética para a Investigação e Ensino—CEIE) of the Faculty of Veterinary Medicine, from the University of Lisbon, Lisbon, Portugal. They were performed by a veterinary doctor as part of a breeding exam, requested by the mare owner’s, who consented on data use for research purposes.

### 2.2. Animals

#### 2.2.1. Experiment 1

This study was carried out in the breeding season (from May to July) on a group of 42 Lusitano mares, ranging from 3 to 23 years old, from different stud farms, located in Portugal (Ribatejo and Alentejo regions). All animals were kept outdoors and maintained on pasture and had free access to water. Mares’ internal genitalia (ovaries, uterus, and cervix) were examined by transrectal ultrasonography (Sonovet 600; rectal linear probe 7.5 MHz), to assess their reproductive status at the time of endometrial biopsy and blood collection. During the breeding season, some of the mares used in this experiment changed owners and location, and some were not used for reproduction. Therefore, follow-up on fertility outcome was not possible. The age of the mares in category I ranged from 3 to 4 years (mean 3.5 ± 0.18; n = 13), in category IIA from 3 to 19 years (5.9 ± 2.01; n = 8), in category IIB from 8–17 years (11.6 ± 1.28; n = 8) and in category III from 10–24 years (mean 16.3 ± 1.26; n = 13). None of the mares had foaled recently. The endometrial biopsy was withdrawn from the uterine body and in the estrous cycle before the first insemination of the breeding season. Endometrial biopsies were collected with a biopsy alligator jaw forceps (ref. 141965; Kruuse, Langeskov, Denmark). Immediately after endometrial biopsy procurement, tissue was divided into two portions: one piece was immersed in 4% buffered formaldehyde solution, and the other one in RNAlater^®^ (R0901; Sigma-Aldrich, St. Louis, MO, USA) that was further kept at −80 °C.

#### 2.2.2. Experiment 2

Experiment 2 was performed, after accomplishment of experiment 1, and on different mares. Therefore, only serum COL3 was assessed, based on the results from experiment 1. In this experiment, 50 Lusitano cyclic mares (27 in follicular phase and 23 in luteal phase) ranging from 3 to 25 years old, from the same stud farm, located in Portugal (Ribatejo region), were used. All animals were kept outdoors and maintained on pasture and had free access to water. None of the mares had recently foaled or had a foal at foot. Mares’ internal genitalia (ovaries, uterus, and cervix) were examined by transrectal ultrasonography (Sonovet 600; rectal linear probe 7.5 MHz), to confirm their reproductive status at the time of blood collection. Insemination of those mares was performed afterwards, during estrus, with fresh semen from different stallions with proven fertility. Since uterine biopsy was not performed in most of the mares, it was not possible to obtain data on endometrial category. Mares were classified as fertile (n = 25; n = 12 in luteal phase; n = 13 in follicular phase) when a gestation diagnosis was positive and confirmed by ultrasonography at 60 days post insemination. Mares were classified as infertile (n = 25; n = 11 in luteal phase; n = 14 in follicular phase) when a negative gestation diagnosis was made, after at least 3 attempts (cycles) of insemination. The age of the fertile mares ranged from 3 to 21 years (7 ± 0.95), and from 5 to 25 years (14.7 ± 1.2) in the infertile mares.

### 2.3. Blood Sampling and Processing

Jugular venous blood was collected from all mares (experiment 1 and experiment 2), at the time of ultrasound examination, into a dry vacutainer, coated with silica as the clot activator (BD Vacutainer^®^ 367896; Becton Dickinson, Winnersh, UK). Blood was centrifuged at 1540× *g* for 10 min and serum was separated, aliquoted, and stored at −80 °C, until COL and hydroxyproline assays were performed.

### 2.4. Endometrium Biopsies Processing

Formaldehyde-fixed endometrium biopsy (experiment 1) was embedded in paraffin, and 4µm-thick histological sections were stained with hematoxylin (05-06014E; Bio-Optica, Milan, Italy) and eosin (HT1103128; Sigma-Aldrich) and were observed by light microscopy (Leica DM500) at 400× magnification. Equine endometrial biopsies histopathological features, such as the extent of inflammation and/or fibrosis, were the grounds for Kenney and Doig’s classification [19]. Therefore, the endometrium was classified, as follows: category I—when the endometrium presented a normal histology or with very mild, focal inflammation or fibrosis; category IIA—when there was mild to moderate inflammation and/or multifocal fibrosis with 1–3 layers of fibroblasts surrounding glands, or less than 2 fibrotic nests per 5 mm linear field; category IIB—when there was moderate inflammation and/or multifocal–diffuse fibrosis with 4 or more layers of fibroblasts surrounding the endometrial glands or 2–4 fibrotic nests per 5 mm linear field; category III— when there was severe inflammation and/or extensive fibrosis with 5 or more fibrotic nests per 5 mm linear field [19]. The endometrial biopsies were assigned to category I (n = 13; 6 in luteal phase and 7 in follicular phase), to category IIA (n = 8; 4 in luteal and 4 in follicular phase), to category IIB (n = 8; 4 in luteal and 4 in follicular phase) or to category III (n = 13; 6 in luteal phase and 7 in follicular phase).

### 2.5. Collagen Determination

Quantification of COL was performed in endometrial biopsy tissue, kept in RNAlater^®^ (experiment 1) and in blood serum (experiments 1 and 2). For quantification of COL in endometrial tissue, the Enzyme-Linked Immunosorbent Assay Kit for Collagen Type 1, COL1 (CEA571Eq; Cloud-Clone Corp., Katy, TX 77494, USA) was used, and for Collagen Type III, COL3 trimer form (CEA176Eq; Cloud-Clone Corp., Katy, TX 77494, USA) was used. Endometrial tissue was macerated and processed according to the manufacturer’s instructions. Briefly, 20 mg of endometrium were homogenized with a lysing solution (1 mL solution/20–50 mg of tissue) and macerated using the TissueLyser II (QIAGEN GmbH, 40724 Hilden, Germany) for 4 cycles of 30 s each, at 75 Hz. After that, cell disruption was performed using an ultrasonic homogenizer (Bandelin Sonopuls, Berlin, Germany) until the solution was clear. Then, centrifugation was performed (10,000× *g* for 5 min). The supernatant was collected, and the assay was performed according to the manufacturer’s kit. The reaction was developed using the tetramethyl benzidine reaction (TMB) substrate, and the absorbance was read using a microplate reader (FLUOstar Optima, BMG LabTech; Baden-Württemberg, Germany) at the wavelength of 450 nm. The concentrations of COL1 and COL3 in each sample were calculated using the standard curve. The detection limit of COL1 and COL3 were 7.15 ng/mL and 4.98 ng/mL, respectively. For each COL type, all samples were run in a single assay. For COL1, the standard curve ranged from 18.52 to 1500 ng/mL, and the intra-assay coefficient of variation (CV) was 8.9%. For COL3, the standard curve ranged from 12.35 ng/mL to 1000 ng/mL, and the intra-assay CV was 6.7%. Protein was extracted from the endometrial tissues as described in [37], and quantification of total protein was performed by Bradford (5000006; Bio-Rad, Hercules, CA, USA) method. Concentrations of COL1 or COL3 were expressed in nanogram (ng) per microgram (μg) of total endometrial protein. Quantification of COL was also assessed in serum of all mares (experiments 1 and 2), using the same ELISAs for COL1 and COL3, as referred to above for the endometrium, and following the manufacturer’s protocol. Serum samples were used directly with no need to undergo the same procedures for tissue samples. Samples were run in duplicate in a single assay. In experiment 1, the intra-assay CVs were 8.8% and 6.9% for serum COL1 and COL3, respectively. In experiment 2, the intra-assay CV was 8.7% for serum COL3. Serum concentrations of COL1 or COL3 were expressed as ng per mL.

### 2.6. Hydroxyproline Determination

Hydroxyproline content was measured in the endometrial tissue (experiment 1) and in the serum (experiments 1 and 2) using Hydroxyproline Colorimetric Assay Kit (K555-100; Biovision, Milpitas, CA, USA). As hydroxyproline is a major component of collagen and comprises about 13.5% of its amino acid composition [38,39], it was used in this study to evaluate total COL concentration in mare endometrium and serum. Briefly, 10 mg of endometrium tissue was homogenized with 100 µL dH20 (100 µL dH20/10 mg of tissue) and macerated using the TissueLyser II (QIAGEN GmbH; 40724 Hilden; Germany) for 4 cycles of 30 s, at 75 Hz. After that, 100 µL of HCL (1 µL HCL/1 µL of sample) were added. For serum samples, 250 µL was used and equal volume of HCL was added. The samples were then placed in a heater, at 119 °C, for 3 h. Afterwards, 4 mg of activated charcoal/100 mL of sample (102186; Sigma-Aldrich; Merck KGaA; St. Louis, MO, USA) was added to the serum samples, as a purification process, and then vortexed, and centrifuged. For each sample of serum or tissue, 10 µL was used, and the protocol was carried out following the manufacturer’s instructions. The hydroxyproline content in tissue and serum samples was quantified colorimetrically by using the chloramine T method, according to the manufacturers protocol, and absorbance was measured at 560 nm, using a microplate reader (FLUOstar Optima, BMG LabTech; Baden-Württemberg, Germany). Hydroxyproline concentration was quantified using a standard curve of high purity hydroxyproline and expressed as ng of hydroxyproline per µg of total endometrial protein or as ng per µL of serum.

The endometrial concentrations of COL1, COL3 or hydroxyproline were expressed as ng/ug of total protein to normalize the parameters assessed in the tissues, as there are no reference values for total protein for the equine endometrium. It is known that in blood, there is a small physiological variation of total protein values. Therefore, serum concentrations of COL1, COL3 or hydroxyproline were expressed in ng/mL, as reference values for equine serum total protein are known.

### 2.7. Statistical Analysis

To establish whether data were normally distributed, variables were tested with Shapiro–Wilk test in both experiment 1 and 2. In experiment 1, to detect differences in COL1 concentration between endometrial categories, one way analysis of variance (ANOVA) followed by Tukey’s multiple comparisons test were performed. Kruskal–Wallis followed by Dunn’s multiple comparisons test were used to detect differences between age, endometrial or serum COL3 concentrations, and endometrial categories. Mann–Whitney test was used to assess differences in the following: (i) concentration of serum COL1, endometrial or serum hydroxyproline among endometrial category; (ii) COL1 or COL3 concentration in serum, or endometrial hydroxyproline and age groups (3–9 years vs. over 9 years). Unpaired *t*-test was performed to evaluate putative differences in COL1 or COL3 concentrations in endometrial tissue and age groups (3–9 years vs. over 9 years) and in hydroxyproline concentration in serum and age groups (3–9 years vs. over 9 years). Spearman correlation was used to assess the following relationships: between endometrial category and (i) age, (ii) endometrial and serum COL1, COL3, and hydroxyproline; among age and (i) endometrial COL1, COL3 and hydroxyproline, or (ii) serum COL1 and COL3. Spearman correlation was also performed to assess the relationship between endometrial and serum concentrations of (i) COL1, (ii) COL3, and (iii) hydroxyproline. Pearson correlation was performed to evaluate the degree of association between age and serum hydroxyproline. In experiment 2, unpaired *t*-test was performed to evaluate differences in COL 3 serum concentration between (i) fertile and infertile mares, and between (ii) age of the mares. Spearman correlation was performed to assess relationship between serum COL3 concentration and (i) fertility, and (ii) age of the mares. The area under receiver operating characteristic curves was determined for COL3, and sensitivity and specificity were calculated. In experiment 1, category I endometrium (healthy) was compared with endometria with degenerative/fibrotic changes (category IIA, IIB, and III), category I and IIA with IIB and III, and severe endometriosis (category III) with all other categories. In experiment 2, fertile mares were compared with infertile mares. The statistical analyses were performed by GraphPAD PRISM (Version 6.01, GraphPad Software, San Diego, CA, USA). Significance was defined as *p* < 0.05. Results were expressed as mean ± standard error of the mean (SEM).

## 3. Results

### 3.1. Experiment 1

**Figure 1:**
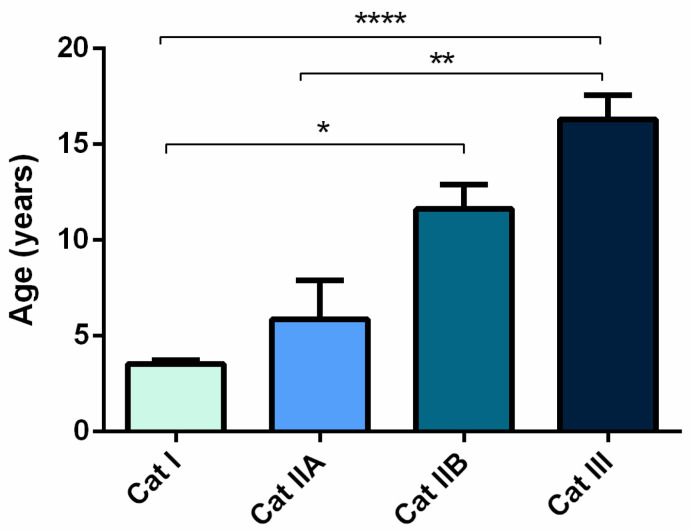
Age of the mares (mean ± SEM) with endometria assigned to different Kenney and Doig’s histopathological categories as I, IIA, IIB or III. Asterisks indicate statistical differences (* *p* < 0.05, ** *p* < 0.01, **** *p* < 0.0001).

The severity of fibrosis in mares’ endometrial biopsies histopathologically graded [19], increased with age (*p* < 0.0001; Figure 1). A very strong correlation was found between mares’ age and endometrial category (ρ = 0.8033; *p* < 0.0001).

**Figure 2:**
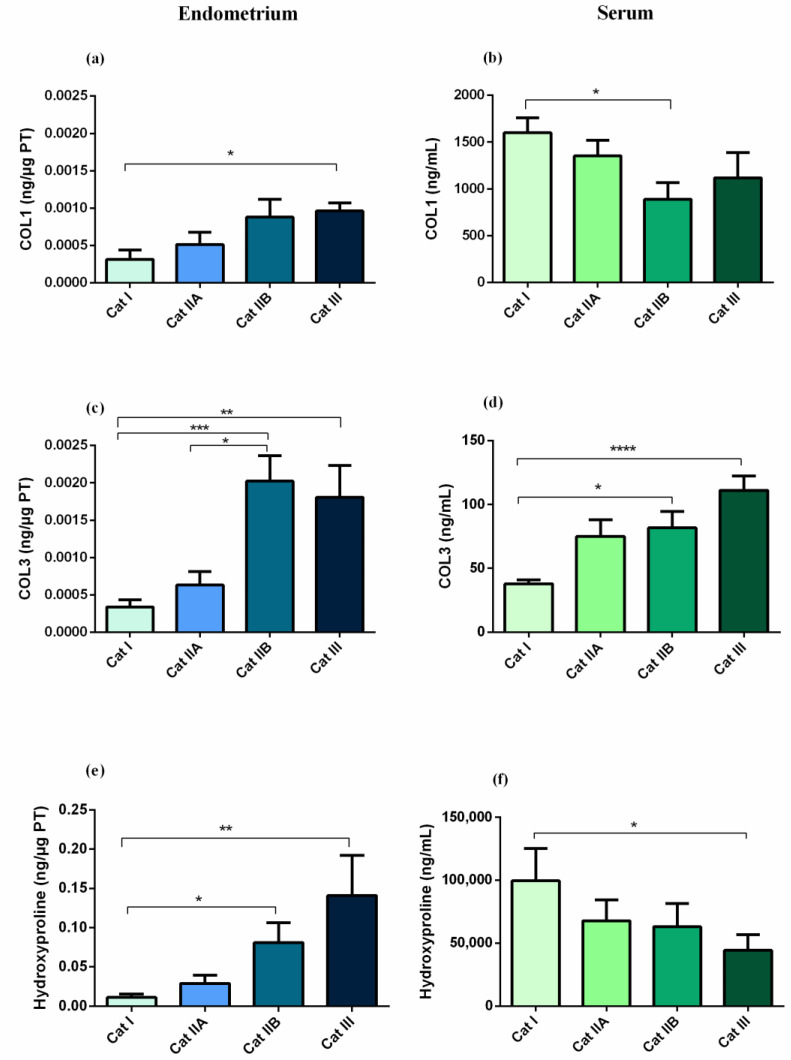
Effect of equine endometrial category (category I, IIA, IIB or III) on the concentration of (**a**) type I collagen (COL1) in endometrial tissue, (**b**) COL1 in serum, (**c**) type III collagen (COL3) in endometrial tissue, (**d**) COL3 in serum, (**e**) hydroxyproline in endometrial tissue, and (**f**) hydroxyproline in serum. Bars represent mean ± SEM. Asterisks indicate significant differences between endometrial categories (* *p* < 0.05; ** *p* < 0.01; *** *p <* 0.001; **** *p* < 0.0001).

**Figure 3:**
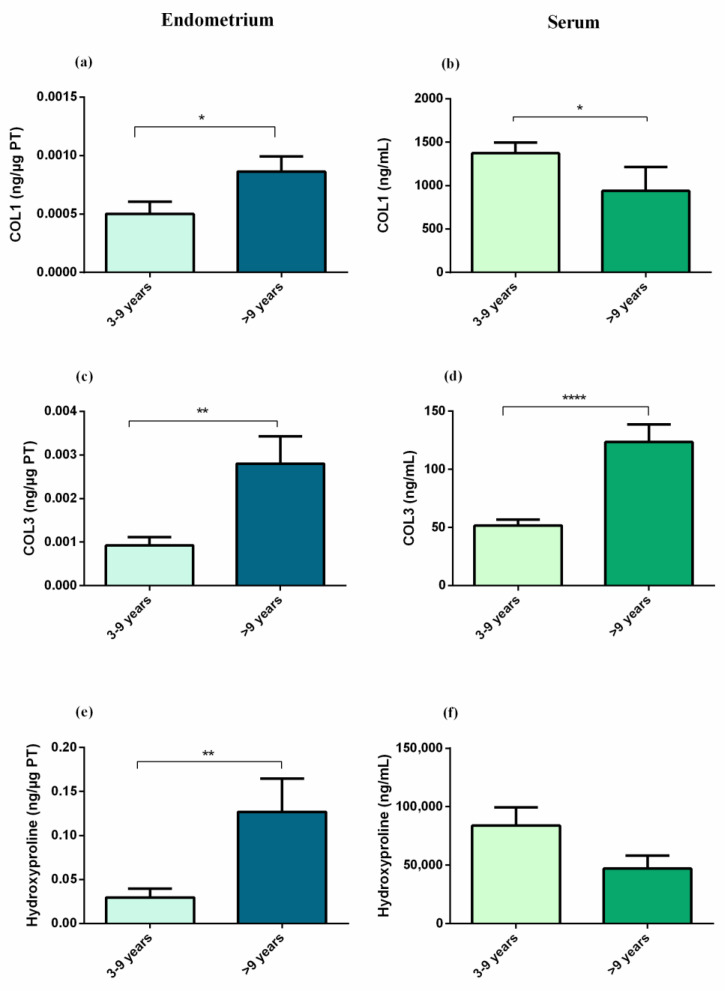
Effect of mares age (3 to 9 years old vs. over 9 years old) on the concentrations of (**a**) type I collagen (COL1) in endometrial tissue, (**b**) COL1 in serum, (**c**) type III collagen (COL3) in endometrial tissue, (**d**) COL3 in serum, (**e**) hydroxyproline in endometrial tissue, and (**f**) hydroxyproline in serum. Bars represent mean ± SEM. Asterisks indicate significant differences between age groups (* *p* < 0.05; ** *p* < 0.01; **** *p* < 0.0001).

The COL1 protein concentration in the endometrial biopsy was higher in category III, when compared to category I (Figure 2a, *p* < 0.05). A moderate correlation was found between endometrial category and endometrial COL1 (ρ = 0.6024; *p* < 0.001). However, in serum, COL1 concentration was higher in category I, healthy endometrium, with respect to category IIB (Figure 2b)**,** and a low negative correlation between endometrial category and serum COL1 was observed (ρ = −0.42; *p* < 0.05). Regarding COL3 in the tissue (Figure 3c), it was higher in category IIB (*p* < 0.001), and III (*p* < 0.01), when compared to category I, and higher in category IIB, when compared to category IIA (*p* < 0.05). A moderate positive correlation was obtained between endometrial category and COL3 in the endometrial tissue (ρ = 0.6360; *p* < 0.0001). The high concentration of COL3 in category IIB, and III endometria was consistent with serum concentration, which was also higher in category IIB (*p* < 0.05)**,** and III (*p* < 0.0001), with respect to category I (Figure 2d). There was a strong correlation between endometrial category and serum COL3 (ρ = 0.7048; *p* < 0.0001). A moderate correlation between endometrial COL3 and serum COL3 (ρ = 0.55; *p* < 0.001) was also observed. Hydroxyproline tissue concentration was elevated in category IIB and III, when compared to category I (*p* < 0.05) (Figure 2e), while in serum the concentration was higher in category I than in category III (*p* < 0.05) (Figure 2f). A positive correlation was observed between endometrial category and hydroxyproline concentration in endometrial tissue (ρ = 0.6770; *p* < 0.001), but no correlation was observed for serum. No correlation was found between endometrial and serum concentrations of COL1 or hydroxyproline. No differences were found between estrous cycle phases (Figure S1).

Mares were also analyzed by age group. When mares were assigned to two different age groups (22 mares, 3–9 years old; 20 mares, over 9 years old), COL1 concentration in endometrium was the highest in the oldest mares, but the lowest in their serum (*p* < 0.05; Figure 3a,b). A positive moderate correlation was found between the age of the mares and endometrial COL1 (ρ = 0.45; *p* < 0.05), and a negative low correlation with serum COL1 (ρ = −0.48; *p* < 0.01). Regarding COL3, its concentration was higher both in endometrial tissues (*p* < 0.01), and in serum (*p* < 0.001) in the oldest animals (Figure 3c,d). There was a low correlation between mares age, and endometrial COL3 (ρ = 0.36; *p* < 0.05), and between mares age, and serum COL3 (ρ = 0.37; *p* < 0.05). Hydroxyproline concentration was elevated in endometrial tissues (*p* < 0.05) in the oldest animals (Figure 3e), but not in serum (Figure 3f). A low correlation between age and endometrial hydroxyproline was observed (ρ =0.48; *p* < 0.05), but no correlation was found between age and serum hydroxyproline.

**Figure 4:**
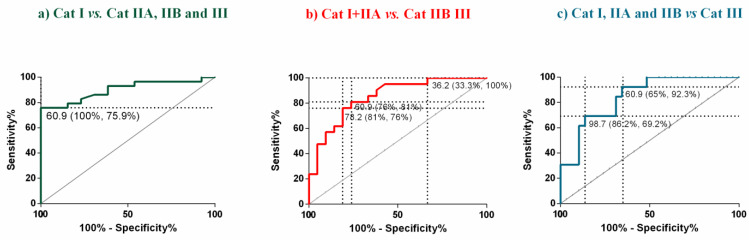
Receiver operating characteristic curves (ROC) of type III collagen for differentiation of the following: (**a**) healthy mares (category I endometrium—Cat I) from mares with endometrial degenerative/fibrotic lesions (Cat IIA, IIB, and III); (**b**) Cat I + IIA endometrium from IIB + III endometrium; (**c**) severe endometriosis (Cat III) from all the other endometrial categories. Cut-off values are expressed in ng/mL (specificity, sensitivity).

The sensibility and specificity of serum COL3 were also determined (Figure 4). Serum COL3 cut-off value of 60.9 ng/mL allowed the differentiation of healthy mares (category I) from mares with endometrial degenerative/fibrotic lesions (categories IIA, IIB, and III), with a specificity of 100% and a sensitivity of 75.9%, and the area under the curve (AUC) was 0.90 (95% confidence interval (CI), 0.81 to 0.99; *p* < 0.0001). The same cut-off value also allowed the differentiation between category I + IIA from IIB + III with a specificity of 76%, a sensitivity of 81%, and an AUC of 0.85 (95% CI, 0.73 to 0.96; *p* = 0.0001), and it allowed the differentiation of category III from all the other endometrial categories with a specificity of 65%, a sensitivity of 92.3%, with an AUC of 0.85 (95% CI, 0.73 to 0.96; *p* = 0.0003). COL1 and hydroxyproline did not prove valid as blood biomarkers of endometrosis.

### 3.2. Experiment 2

**Figure 5:**
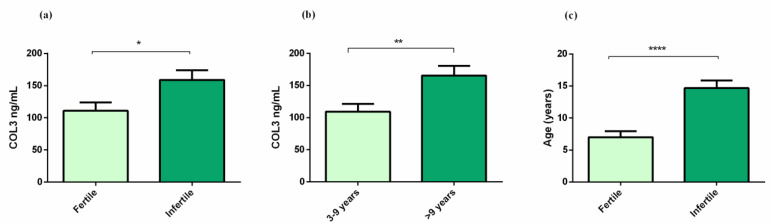
(**a**) Effect of fertility on serum type III collagen (COL3); (**b**) effect of age on serum COL3; (**c**) effect of age on fertility. Bars represent mean ± SEM. Asterisks indicate significant differences between fertility or age groups (* *p* < 0.05; ** *p* < 0.01, **** *p* < 0.0001).

There were higher COL3 serum concentrations in infertile mares when compared to fertile mares (*p* < 0.05; Figure 5a). Higher COL3 in the older mares’ serum (>9 years) was also observed (*p* < 0.01; Figure 5b). There was a low correlation between serum COL3 and fertility of the mares (ρ = 0.35, *p* < 0.05), and no correlation was observed between serum COL3 and mare’s age. Mare’s age was higher in the infertile group (14.68 years ± 1.2), when compared to the fertile group (7 years ± 0.95; *p* < 0.0001) (Figure 5c), and there was a moderate correlation between fertility and the age of the mares (ρ = 0.68, *p* < 0.0001). There were no differences with respect to the phase of the estrous cycle (Figure S2).

**Figure 6:**
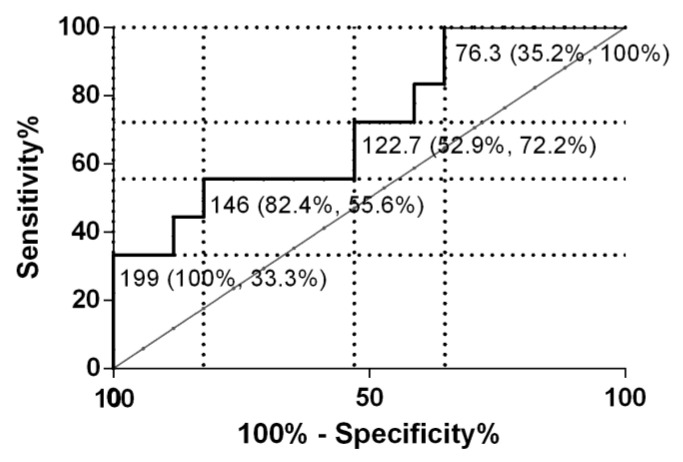
Receiver operating characteristic curves (ROC) of serum type III collagen for differentiation of fertile from infertile mares. Cut-off values are expressed in ng/mL (specificity, sensibility).

The mares were then analyzed separately in each group (fertile and infertile) to assess the age effect (3–9 years old vs. over 9 years old) on serum COL3 concentrations. In both fertile and infertile groups, there were no differences between mare’s age and serum COL3 (Figure S3). Additionally, no correlation was found between age and serum COL3 in both fertile and infertile mares (r = 0.128, *p* > 0.05; and r = −0.009, *p* > 0.05, respectively). The sensibility and specificity of serum COL3 were also determined. Serum COL3 cut-off value of 146 ng/mL allowed the differentiation of fertile from infertile mares, with a specificity of 82.4%, a sensitivity of 55.6%, and the AUC was 0.72 (95% CI, 0.55 to 0.89; *p* = 0.029) (Figure 6).

## 4. Discussion

In humans, many studies are being conducted to find diagnostic and prognostic fibrotic biomarkers for cardiac [40,41], renal [42], and hepatic fibrosis [37,38,43], among many others [32]. Biomarkers may be used for early detection of otherwise subclinical disease, diagnostic assessment of an acute or chronic clinical syndrome, risk stratification of patients with a suspected or confirmed diagnosis, prognosis, and selection of an appropriate therapeutic intervention, and monitoring the response to therapy [41]. As an example, a study in abdominal aortic aneurysm in humans, higher amounts of hydroxyproline, COL1, and COL3 were found in samples of patients compared with healthy controls, and a positive correlation was found between tissue and serum concentrations of COL1, and COL3 [39]. Therefore, we aimed to identify blood biomarkers for the diagnosis of endometrosis and fertility in mares. Uterine biopsy is an invasive procedure and evaluation of blood biomarkers could facilitate the diagnosis of endometrosis, and/or to predict fertility, and provide additional information.

In the present work, up-regulation of COL1 protein production in mare endometrium, as the severity of histopathological lesions increased, according to Kenney and Doig’s classification, was noted, as previously shown [44]. This raise was also seen in COL3 and hydroxyproline concentrations in the endometrium, suggesting that both collagens are being produced when fibrosis increases. Nevertheless, it has been previously described that COL1 fibers are replaced by COL3 fibers with fibrosis, in the equine endometrium, and in several human organs, such as in liver and heart [15,16,17]. However, other studies suggest that COL3 appears to regulate COL1 fibril formation in many human organs [45,46]. In humans and animals [47], COL1 exists in higher amounts, accounting for over 90% of bone protein, being also very high in skin and other soft tissues. Therefore, the use of COL1 as a blood biomarker should be interpreted with caution, as it may be related to pathologies of other organs [33]. In contrast to COL1, the expression of COL3 is restricted to soft tissues, and correlates to the number of myofibroblasts in fibrotic tissue [48]. Consequently, the accuracy for the presence of fibrotic processes in soft tissues is greater for COL3 (and other minor collagens), as compared to COL1 [48]. In fact, type III procollagen peptides have been regarded as good prognostic biomarkers for liver fibrosis in humans [49,50]. However, it should be emphasized that COL3 is not an endometrial specific biomarker, since its plasma elevation can occur due to other organ diseases [51]. The increase in COL1 and COL3 production in the endometrium was substantiated by a raise in hydroxyproline concentration (major component of COL) in tissues with severe endometrosis, and advanced fibrosis (categories IIB and III). Due to the highly restricted distribution of hydroxyproline in collagen and elastin, the hydroxyproline content generally reflects the amount of collagen in samples. In the serum, there was a significant decrease in COL1 between category I and IIB, and in hydroxyproline in category III compared to category I. Since COL1 is the most abundant fibrillar collagen, making up about 95% of the total collagen in animal tissue [47], hydroxyproline in the mare also displayed the same patterns of COL1, both in endometrium and in serum, when endometrosis was present. In addition, no correlation was found between endometrial and serum concentrations of COL1 or hydroxyproline. Thus, neither of them seems viable as a biomarker of endometrial fibrosis, and therefore, they should be ruled out. In our study, only COL3 data suggest it may be related to fibrosis, increasing both in serum and endometrium with fibrosis. Indeed, a moderate correlation between endometrial and serum COL3 concentration was observed, and a strong correlation was noted between endometrial category and serum COL3.

A ROC curve is an established method for evaluating the clinical viability of a biomarker [27]. The sensibility and specificity of COL3 was most effective to differentiate mares from category I from all other categories, when compared to the ability to differentiate category I + category IIA from category IIB + III, or to identify mares with severe endometrosis (category III) among all the other endometrial categories. Serum COL3 was also higher in infertile mares, when compared to fertile mares, and a low positive correlation was observed between serum COL3 and infertile mares. Sensibility and specificity of COL3 was more accurate in differentiating healthy mares from mares with endometrial fibrosis, than for separating fertile mares from infertile mares. This might be explained by the fact that infertility in mares may be due to several causes other than endometrosis, and because both fertile and infertile groups of mares may include mares with different endometrial categories.

Old age has been associated with increased endometrial inflammation, increased embryo-loss rate, and subsequently reduced pregnancy rate in the mare [52]. Although the age factor is not by itself the cause of endometrosis, in this study the endometrial fibrosis assessed by histopathological examination, was higher in older mares (category IIB and III) compared to the youngest ones, and a strong correlation between age and severity of the disease was found. This agrees with previous work [2,7,8,13,53]. Aging of the mares has also been related to increased fibrosis in the oviduct [54], which might be an additional cause of infertility/sub-fertility in this species. Furthermore, there was an increase in both types of collagens under study and hydroxyproline in the endometrium with mares aging, suggesting that the age factor plays an important/significant role and should not be dismissed. Regarding serum concentrations, COL1 was higher, and COL3 was lower in young mares, when compared to older mares, and no differences were found in hydroxyproline. Higher serum COL1 concentrations in younger horses, and lower in older horses, have been previously described [55]. Most of those studies refer to equine musculoskeletal pathologies, and the described high serum COL1 concentrations appear to be ascribed to the ongoing bone formation in young horses. Therefore, serum COL1 is more likely to represent bone and cartilage status than endometrosis. Hence, the age factor may interfere with its use as an endometrial fibrosis biomarker. In the present study, the highest serum COL1 concentrations observed in mares with healthy endometrium (category I), and the lowest ones in mares with endometrial fibrosis, might be explained by the inclusion of younger mares in category I, and of older ones in categories IIB and III. The infertile mares were older than the fertile mares, and a moderate positive correlation was observed between age and infertility. However, no correlation was observed between serum COL3 and mare’s age.

To the best of our knowledge no other studies were carried out on serum COL3, to evaluate endometrial fibrosis and fertility in mares. As stated above, it should be emphasized that COL3 is not an endometrial specific biomarker, and alterations in its concentration can occur due to other organ diseases (51). Therefore, further studies are necessary to study the possible involvement of COL3 in other organs. Additionally, many collagen biomarkers detect a specific type of COL, but do not distinguish between newly formed and older matrix-deposited COL, or between COL formation and degradation [35], as in this study. Hence, the investigation of COL turnover, by determination of its synthesis and degradation products, could give a more precise information regarding the stage of fibrosis, and should be further investigated.

## 5. Conclusions

In conclusion, serum COL3 concentration might be considered as a potential aid for the diagnosis of endometrial fibrosis and fertility prognosis in the mare. In contrast, COL1 and hydroxyproline did not prove to be effective as putative biomarkers of endometrial fibrosis in this species. Although it is very unlikely a single blood biomarker could replace a histopathological evaluation, serum COL3 may have clinical applications. As such, it may be used to evaluate a group of mares as possible recipients in embryo transfer programs, where performing endometrial biopsies of several mares is not feasible.

## Data Availability

The data that support the findings of this study are available from the corresponding author upon reasonable request.

## Supplementary Materials

The following are available online at https://www.mdpi.com/article/10.3390/ani12141854/s1, Figure S1: Effect of equine estrous cycle phases (Folicular phase—FP and Luteal phase—LP) on the concentration of (a) type I collagen (COL1) in endometrial tissue; (b) COL1 in serum; (c) type III collagen (COL3) in endometrial tissue; (d) COL3 in serum; (e) hydroxyproline in endometrial tissue; and (f) hydroxyproline in serum. Bars represent mean ± SEM. Figure S2: Effect of equine estrous cycle phases (Folicular phase—FP and Luteal phase—LP) on the concentration of serum COL3. Bars represent mean ± SEM. Figure S3: Effect of mares age (3 to 9 years old vs. over 9 years old) on the serum concentrations of type III collagen (COL3) in (a) fertile mares and (b) infertile mares. Bars represent mean ± SEM.

## References

[1] Kenney R.M. (1993). The aetiology, diagnosis and classification of chronic degenerative endometritis (CDE) (endometrosis). Proceedings of the John P. Hughes International Workshop on Equine Endometritis. Davis, California, August 1992. Equine Vet. J..

[2] Schoon H.A., Schoon D., Klug E. (1992). Uterusbiopsien als Hilfsmittel für Diagnose und Prognose von Fertilitätsstörungen der Stute. Pferdeheilkunde.

[3] Schoon H.A., Schoon D., Klug E. (1997). Die Endometriumbiopsie bei der Stute im klinisch-gynäkologischen Kontext. Pferdeheilkun-de.

[4] Gray C.A., Bartol F.F., Tarleton B.J., Wiley A.A., Johnson G.A., Bazer F.W., Spencer T.E. (2001). Developmental biology of uterine glands. Biol. Reprod..

[5] Schoon H.A., Schoon D. (2003). The category I mare (Kenney and Doig 1986): Expected foaling rate 80–90%-Fact or fiction?. Pferde-heilkunde.

[6] Ebert A., Schoon D., Schoon H.A., Rackwitz R., Pees M., Aschenbach J.R., Gäbel G. (2014). Age-related endometrial alterations in mares-biopsy findings of the last 20 years. Leipziger Blaue Hefte, 7th Leipzig Veterinary Congress, 8th International Conference on Equine Reproductive Medicine.

[7] Ricketts S.W., Alonso S. (1991). The effect of age and parity on the development of equine chronic endometrial disease. Equine Vet.-J..

[8] Hoffmann C., Ellenberger C., Mattos R.C., Aupperle H., Dhein S., Stief B., Schoon H.-A. (2009). The equine endometrosis: New insights into the pathogenesis. Anim. Reprod. Sci..

[9] Aresu L., Benali S., Giannuzzi D., Mantovani R., Castagnaro M., Falomo M.E. (2012). The role of inflammation and matrix metallo-proteinases in equine endometriosis. J. Vet. Sci..

[10] Raila G. (2020). Zur Pathogenese der Endometrose der Stute. Morphologisch-Funktionelle Untersuchungen. Ph.D. Thesis.

[11] Evans T.J., Miller M.A., Ganjam V.K., Niswender K.D., Ellersieck M.R., Krause W.J., Youngquist R.S. (1998). Morphometric analysis of endometrial periglandular fibrosis in mares. Am. J. Vet. Res..

[12] Walter I., Handler J., Reifinger M., Aurich C. (2001). Association of endometriosis in horses with differentiation of periglandular myofibroblasts and changes of extracellular matrix proteins. Reproduction.

[13] Hoffmann C., Bazer F.W., Klug J., Aupperle H., Ellenberger C., Schoon H.A. (2009). Immunohistochemical and histochemical identifi-cation of proteins and carbohydrates in the equine endometrium expression patterns for mares suffering from endometrosis. Theriogenology.

[14] Guyot C., Lepreux S., Combe C., Doudnikoff E., Bioulac-Sage P., Balabaud C., Desmoulière A. (2006). Hepatic fibrosis and cirrhosis: The (myo)fibroblastic cell subpopulations involved. Int. J. Biochem Cell Biol..

[15] Masseno A.P.B. (2012). Avaliação da Fibrose Endometrial e dos Miofibroblastos na Endometroses Ativa e Inativa das Éguas. Ph.D. Thesis.

[16] Martinez-Hernandez A., Rubin E., Faber J.L. (1999). Repair, regeneration and fibrosis. Pathology.

[17] Bochsler P.N., Slauson D.O., Slauson D.O., Cooper B.J. (2002). Inflammation and repair of tissue. Mechanisms of Disease: A Textbook of Comparative General Pathology.

[18] Kenney R.M. (1978). Cyclic and pathologic changes of the mare endometrium as detected by biopsy, with a note on early embryonic death. J. Am. Vet. Med. Assoc..

[19] Kenney R.M., Doig P.A., Morrow D.A. (1986). Equine endometrial biopsy. Current Therapy in Theriogenology 2: Diagnosis, Treatment, and Prevention of Reproductive Diseases in Small and Large Animals.

[20] Flores J.M., Rodriguez A., Sanchez J., Gomez-Cuetara C., Ramiro F. (1995). Endometrosis in Mares: Incidence of Histopathological Alterations. Reprod. Dom. Anim..

[21] Ricketts S.W., Barrelet A. (1997). A retrospective review of the histopathological features seen in a series of 4241 endometrial biopsy samples collected from UK Thoroughbred mares over a 25 year period. Pferdeheilkunde.

[22] Katkiewicz M., Witkowski M., Zajac S. (2007). Endometrial biopsy of mares: Visualization of healthy and diseased structure. Med. Weter.

[23] Zajac S., Katkiewicz M., Witkowski M., Boryczko Z., Pawlak M. (2008). Endometrosis in mares. Med. Weter.

[24] Schlafer D.H. (2007). Equine endometrial biopsy: Enhancement of clinical value by more extensive histopathology and application of new diagnostic techniques?. Theriogenology.

[25] Snider T.A., Sepoy C., Holyoak G.R. (2011). Equine endometrial biopsy reviewed: Observation, interpretation, and application of histopathologic data. Theriogenology.

[26] Hanada M., Maeda Y., Oikawa M.A. (2014). Histopathological characteristics of endometrosis in thoroughbred mares in Japan: Results from 50 necropsy cases. J. Equine Sci..

[27] Luo Y., Oseini A., Gagnon R., Charles E., Sidik K., Vincent R., Collen R., Idowu M., Contos M., Mirshahi F. (2018). An evaluation of the collagen fragments related to fibrogenesis and fibrolysis in nonalcoholic steatohepatitis. Sci. Rep..

[28] Frisbie D.D., Ray C.S., Ionescu M., Poole A.R., Chapman P.L., McIlwraith C.W. (1999). Measurement of synovial fluid and serum concentrations of the 846 epitope of chondroitin sulfate and of carboxy propeptides of type II procollagen for diagnosis of os-teochondral fragmentation in horses. Am. J. Vet. Res..

[29] Laverty S., Ionescu M., Marcoux M., Boure L., Doize B., Poole A.R. (2000). Alterations in cartilage type-II procollagen and aggrecan contents in synovial fluid in equine osteochondrosis. J. Orthop. Res..

[30] Billinghurst R.C., Brama P.A.J., van Weeren P.R., McIlwraith C.W. (2004). Evaluation of serum concentrations of biomarkers of skeletal metabolism and results of radiography as indicators of severity of osteochondrosis in foals. Am. J. Vet. Res..

[31] Frisbie D.D., Al-Sobayil F., Billinghurst R.C., Kawcak C.E., McIlwraith C.W. (2008). Changes in synovial fluid and serum biomarkers with exercise and early osteoarthritis in horses. Osteoarthr. Cartil..

[32] Gressner O.A., Weiskirchen R., Gressner A.M. (2007). Biomarkers of hepatic fibrosis, fibrogenesis and genetic pre-disposition pending between fiction and reality. J. Cell Mol. Med..

[33] Karsdal M.A., Daniels S.J., Holm Nielsen S., Bager C., Rasmussen D.G.K., Loomba R., Surabattula R., Villesen I.F., Luo Y., Shevell D. (2020). Collagen biology and non-invasive biomarkers of liver fibrosis. Liver Int..

[34] Bruckner P. (2010). Suprastructures of extracellular matrices: Paradigms of functions controlled by aggregates rather than molecules. Cell Tissue Res..

[35] Karsdal M.A., Woodworth T., Henriksen K., Maksymowych W.P., Genant H., Vergnaud P., Christiansen C., Schubert T., Qvist P., Schett G. (2011). Biochemical markers of ongoing joint damage in rheumatoid arthritis--current and future applications, limitations and opportunities. Arthritis Res. Ther..

[36] Neto da Silva A.C., Costa A.L., Teixeira A., Alpoim-Moreira J., Fernandes C., Fradinho M.J., Rebordão M.R., Silva E., Ferreira da Silva J., Bliebernicht M. (2022). Collagen and Microvascularization in Placentas From Young and Older Mares. Front. Vet. Sci..

[37] Rosenberg W.M., Voelker M., Thiel R., Becka M., Burt A., Schuppan D., Hubscher S., Roskams T., Pinzani M., Arthur M.J. (2004). European Liver Fibrosis Group. Serum markers detect the presence of liver fibrosis: A cohort study. Gastroenterology.

[38] Tanwar S., Trembling P.M., Hogan B.J., Srivastava A., Parkes J., Harris S., Grant P., Nastouli E., Ocker M., Wehr K. (2017). Noninvasive markers of liver fibrosis: On-treatment changes of serum markers predict the outcome of antifibrotic therapy. Eur. J. Gastroenterol. Hepatol..

[39] Metschl S., Reutersberg B., Maegdefessel L., Eckstein H., Pelisek J. (2019). Collagen Type I and III in Serum of Patients with Abdominal Aortic Aneurysm: Potential Biomarker of Risk Stratification?. Arterioscler. Thromb. Vasc. Biol..

[40] Ong K.L., Chung R.W.S., Hui N., Festin K., Lundberg A.K., Rye K.A., Jonasson L., Kristenson M. (2020). Usefulness of Certain Protein Biomarkers for Prediction of Coronary Heart Disease. Am. J. Cardiol..

[41] Zannad F., Rossignol P., Iraqi W. (2010). Extracellular matrix fibrotic markers in heart failure. Heart Fail. Rev..

[42] Ix J.H., Biggs M.L., Mukamal K., Djousse L., Siscovick D., Tracy R., Katz R., Delaney J.A., Chaves P., Rifkin D.E. (2015). Urine Collagen Fragments and CKD Progres-sion-The Cardiovascular Health Study. J. Am. Soc. Nephrol..

[43] Soylemezoglu O., Wild G., Dalley A.J., MacNeil S., Milford-Ward A., Brown C.B., el Nahas A.M. (1997). Urinary and serum type III collagen: Markers of renal fibrosis. Nephrol. Dial. Transplant..

[44] Lunelli D., Cirio S.M., Leite S.C., Camargo C.E., Kozicki L.E. (2013). Collagen types in relation to expression of estradiol and pro-gesterone receptors in equine endometrial fibrosis. Adv. Biosci. Biotechnol..

[45] Liu X., Wu H., Byrne M., Krane S., Jaenisch R. (1997). Type III collagen is crucial for collagen I fibrillogenesis and for normal cardi-ovascular development. Proc. Natl. Acad. Sci. USA.

[46] Kuivaniemi H., Tromp G. (2019). Type III collagen (COL3A1): Gene and protein structure, tissue distribution, and associated diseases. Gene.

[47] Miller E.J., Gay S. (1987). The collagens: An overview and update. Methods Enzymol..

[48] Badid C., Vincent M., McGregor B., Melin M., Hadj-Aissa A., Veysseyre C., Hartmann D.J., Desmouliere A., Laville M. (2000). My-cophenolate mofetil reduces myofibroblast infiltration and collagen III deposition in rat remnant kidney. Kidney Int..

[49] Nielsen M.J., Veidal S.S., Karsdal M.A., Ørsnes-Leeming D.J., Vainer B., Gardner S.D., Hamatake R., Goodman Z.D., Schuppan D., Patel K. (2015). Plasma Pro-C3 (N-terminal type III collagen propeptide) predicts fibrosis progression in patients with chronic hepatitis C. Liver Int..

[50] Leeming D.J., Dolman G., Nielsen M.J., Karsdal M.A., Patel K., Irving W., Guha I.N. (2016). True collagen type III formation (Pro-C3) is predictive of outcome in HCV patients with advanced liver fibrosis with in the trent study. J. Hepatol..

[51] Gressner O., Weiskirchen R., Gressner A. (2007). Biomarkers of liver fibrosis: Clinical translation of molecular pathogenesis or based on liver-dependent malfunction tests. Clin. Chim. Acta.

[52] Carnevale E.M., Ginther O.J. (1992). Relationship of age to uterine function and reproductive efficiency in mares. Theriogenology.

[53] Rebordão M.R., Amaral A., Lukasik K., Szóstek-Mioduchowska A., Pinto-Bravo P., Galvão A., Skarzynski D.J., Ferreira-Dias G. (2019). Impairment of the antifibrotic prostaglandin E2 pathway may influence neutrophil extracellular traps-induced fibrosis in the mare endometrium. Domest. Anim. Endocrinol..

[54] Pinto-Bravo P., Rebordão M.R., Amaral A., Fernandes C., Cuello C., Parrilla I., Martínez E., Roberto da Costa R.P., Skarzynski D.J., Ferreira-Dias G. (2018). Is mare endometrosis linked to oviduct fibrosis?. Pferdeheilkunde Equine Med..

[55] Price J.S., Jackson B., Eastell R., Goodship A.E., Blumsohn A., Wright I., Stoneham S., Lanyon L.E., Russell R.G. (1995). Age related changes in biochemical markers of bone metabolism in horses. Equine Vet. J..

